# Pharmacist Peer‐Led Teaching Enhances Medical Undergraduate Prescribing: A Mixed‐Methods Study

**DOI:** 10.1111/tct.70192

**Published:** 2025-09-09

**Authors:** Zain Mohammed, Mohammed Sarwar Shah, Imtanaan Abbas, Nabeel Hussain, Shehzar Shah, Saira Chowdry, Shyam Balasubramanian, Kate Owen

**Affiliations:** ^1^ Warwick Medical School Coventry UK

## Abstract

**Background:**

Prescribing is a high‐stakes clinical task where newly qualified doctors frequently report low confidence, with national data highlighting persistent error rates. Medical schools face logistical and staffing barriers in delivering high‐quality, simulation‐based prescribing education. Peer‐led, interprofessional teaching, particularly by pharmacists, may offer a scalable solution in this context.

**Approach:**

This is the first study to evaluate pharmacist peer‐led prescribing education using a mixed‐methods framework. We designed and implemented a pharmacist peer‐led prescribing programme for final‐year medical students at Warwick Medical School. Peer tutors were final‐year medical students with pharmacy backgrounds. Participants were recruited from the final year cohort (*n* = 74) and were split randomly across two groups, receiving teaching in a crossover format at different intervals. Teaching focused on calculations, high‐risk drugs and prescribing in clinical scenarios. Mixed‐methods evaluation included simulated assessments, confidence questionnaires, national PSA performance and semi‐structured interviews. Quantitative data were analysed using non‐parametric tests, and qualitative data were thematically analysed using Braun and Clarke's framework.

**Evaluation:**

Seventy‐four students participated. Simulated prescribing assessment scores significantly improved within both groups (*p* < 0.001), with large effect sizes and strong domain‐level gains (e.g., calculations +38.1%, data interpretation +37.2%). PSA scores and pass rates were higher among participants. Confidence improved across all domains (*p* < 0.001). Thematic analysis revealed four key enablers: specialist peer insights, interactive delivery, psychological safety and curriculum alignment.

**Implications:**

This novel pharmacist‐led, peer‐teaching model improved prescribing skills, confidence and interprofessional awareness. Now adopted locally and nationally, it offers a transferable, low‐cost framework for embedding peer‐led simulation into prescribing education.

## 1 Background

Prescribing is a fundamental clinical skill for newly qualified doctors. In the United Kingdom, the Prescribing Safety Assessment (PSA) is a prerequisite to progression beyond Foundation Year 1, with many NHS trusts requiring its completion before prescribing rights are granted [1, 2]. The EQUIP study found 10% of hospital prescriptions involved errors, with foundation doctors responsible for a significant share [3]. These mistakes can be clinically and financially costly, estimated at £2.5 billion annually [4]. Medical students report feeling underprepared to prescribe, highlighting a lack of structured teaching, limited prescribing simulation and minimal pharmacist involvement in their training [5, 6].

Simulation‐based prescribing has shown promise in improving knowledge and confidence, but its delivery across UK medical schools is inconsistent, limited by staffing pressures and infrastructure [6, 7]. Pharmacists are well positioned to address curricular gaps, especially in managing complex prescriptions, interpreting drug interactions and effectively using clinical tools such as the BNF [8].

At Warwick Medical School (WMS), a graduate‐entry programme, several students are often qualified pharmacists. This created a unique opportunity to trial a new model of peer‐led teaching delivered by pharmacists to medical student peers. This timely intervention aligns with the 2023 Independent Review of the PSA, which explicitly called for practical, simulation‐based approaches to improve prescribing safety [2].

Pharmacist‐led teaching is gaining recognition for its effectiveness, particularly as pharmacists bring a depth of knowledge in pharmacology, high‐risk medications and drug interactions. Whereas small‐scale studies have shown positive results, most lack rigorous evaluation, control groups or qualitative insight into the learner experience [8, 9, 10]. When these pharmacists are also peers—working alongside students—they bring an added layer of cognitive congruence, which can help make prescribing feel more accessible, relevant and less intimidating [11].

We used a mixed‐methods approach to evaluate the intervention. This allowed us not only to assess changes in prescribing performance and confidence but also to understand how the teaching was experienced and what value the pharmacist peer element added. This study aimed to pilot and evaluate a pharmacist peer‐led prescribing teaching model, examining its feasibility, impact on medical students' prescribing confidence, and performance in both simulated prescribing tasks and the national PSA.

## 2 Approach

This study was conducted at WMS, a graduate‐entry programme where several final‐year students were also qualified pharmacists. A peer‐led teaching intervention was developed to address persistent national concerns around prescribing education. This involved ‘pharmacist peers’ delivering a structured, simulation‐based curriculum aligned with the PSA blueprint. Ethical approval was granted by the Biomedical and Scientific Research Ethics Committee at WMS via course delegated approval in March 2023. Participant informed consent was also undertaken, with signed consent forms completed.

*This involved ‘pharmacist peers’ delivering a structured, simulation‐based curriculum aligned with the PSA blueprint*.

### 2.1 Participants and Study Design

**FIGURE 1:**
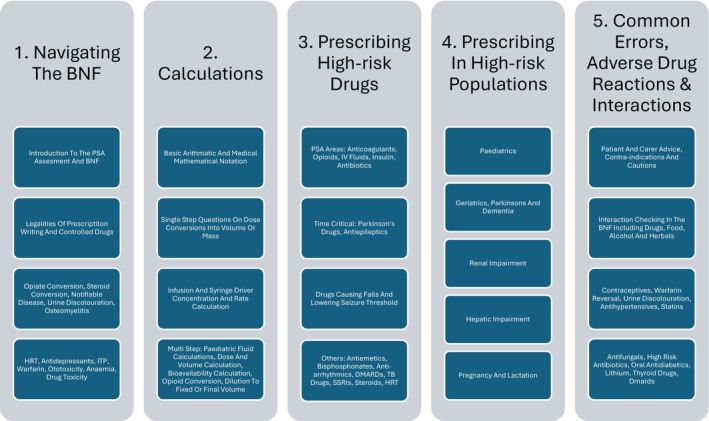
Overview of curriculum content for pharmacist‐led teaching intervention.

All 185 final‐year medical students were invited to participate; 140 expressed interest, with 80 randomly selected using computer‐generated allocation, stratified into Stream A and Stream B (*n* = 40 each). Following six withdrawals, 36 students remained in Stream A and 38 in Stream B. Randomisation was independent, with tutors blinded to participant identities. The intervention consisted of five weekly 90‐min sessions per stream, delivered by qualified pharmacist peer tutors with prior clinical teaching experience. Tutors received standardised training and followed a pre‐approved session plan to ensure consistency. Each tutor rotated across both streams, delivering identical content to mitigate tutor variability. Curriculum and assessment materials were reviewed and validated by prescribing faculty and piloted with a small student group to ensure clarity, appropriate difficulty and relevance. Simulated assessment materials, including MCQs and written prescription scenarios, were developed by pharmacist tutors and refined in collaboration with prescribing leads. These were mapped directly to the national PSA blueprint, reviewed for content validity and piloted with non‐participating students. Free‐text prescribing responses were marked using rubrics based on National PSA guidance. Session content has been described in detail in Figure 1.

### 2.2 Curriculum and Educational Framework

Sessions focused on high‐risk prescribing scenarios, BNF navigation, calculations and prescribing for vulnerable populations. Teaching involved clinical cases, MCQs, simulated prescribing tasks and whiteboard explanations. The programme was informed by key pedagogical principles (summarised in Table 1). Social constructivism underpinned small‐group collaboration; cognitive congruence enabled relatable, peer‐level explanation; adult learning theory supported practical, clinically relevant content; interprofessional learning provided insight into pharmacist perspectives; and simulated assessment theory guided alignment with real‐world prescribing [12, 13, 14, 15, 16].

**TABLE 1:** Educational theory underpinning the pharmacist peer‐led teaching model.

Theoretical principle	Description	Application	Intended outcomes
Social constructivism	Knowledge is constructed through social interaction and dialogue with peers	Delivered through small‐group, interactive, discussion‐based learning	Enhanced student engagement Deeper conceptual understanding via peer collaboration
Cognitive congruence	Peer tutors who are closer in academic level can provide more relatable explanations and foster a comfortable learning environment	All tutors were pharmacist peers from the same final‐year graduate‐entry medicine (GEM) cohort	Improved identification of curricular gaps Promoted a shared sense of journey and empathy between facilitators and learners
Simulated/exam‐style learning	Learning is most effective when anchored in realistic, context‐rich scenarios	Use of clinical case studies that mirrored both real‐life prescribing situations and PSA exam formats	Greater preparedness for real‐world prescribing Increased confidence in managing PSA‐style cases
Adult learning theory	Adult learners are motivated by content that is practical, relevant and applicable to their professional roles	Focus on high‐yield topics, clinically relevant prescribing and high‐risk patient groups	Increased learner motivation Stronger perception of relevance and applicability to clinical practice
Interprofessional learning	Exposure to other professional roles fosters collaboration and understanding within the healthcare team	Sessions led by qualified pharmacists offering expert insight and interprofessional perspectives	Improved understanding of the pharmacist's role Encouraged collaboration within the multidisciplinary team (MDT)

*Teaching involved clinical cases, MCQs, simulated prescribing tasks and whiteboard explanations*.

### 2.3 Assessment and Evaluation

A mixed‐methods evaluation assessed feasibility, impact and learning gains. Students sat three ‘simulated assessments’, that is, 50‐mark PSA‐style practice assessment (Exam 1: baseline, Exam 2: midpoint, Exam 3: post‐intervention). Stream A completed the course before Exam 2; Stream B completed before Exam 3. Assessments mirrored PSA structure, comprising MCQs and written prescribing scenarios. MCQs were auto‐marked; written prescriptions were scored against a standardised rubric by blinded assessors. Assessments were validated through faculty prescribing leads and pilot testing.

Students who attended the intervention completed pre‐ and post‐course confidence questionnaires using a 5‐point Likert scale mapped to PSA domains and high‐risk drug groups. Students were blinded to their pre‐attendance responses. PSA results from participating students were obtained via university records and compared with non‐participants (students who did not enrol or were not selected). To capture student experiences, nine participants were selected for semi‐structured interviews via computer randomisation. Interviews were transcribed and analysed thematically by two independent coders using Braun and Clarke's framework until thematic saturation.

## 3 Evaluation

### 3.1 Simulated Assessment Performance

A total of 74 participants completed all three simulated prescribing assessments across two teaching streams. Statistical analysis showed robust improvements in prescribing performance, confidence and skills following the pharmacist peer‐led intervention. To assess internal consistency of examinations, Cronbach's alpha was calculated by domain across all exams. Assessment level alpha scores ranged from 0.19 to 0.32. Domain level scores ranged from 0.575 (calculations, Exam 1) to 0.743 (prescribing, Exam 3). These values increased across the three assessments. Full results are available in Appendix 1.

**FIGURE 2:**
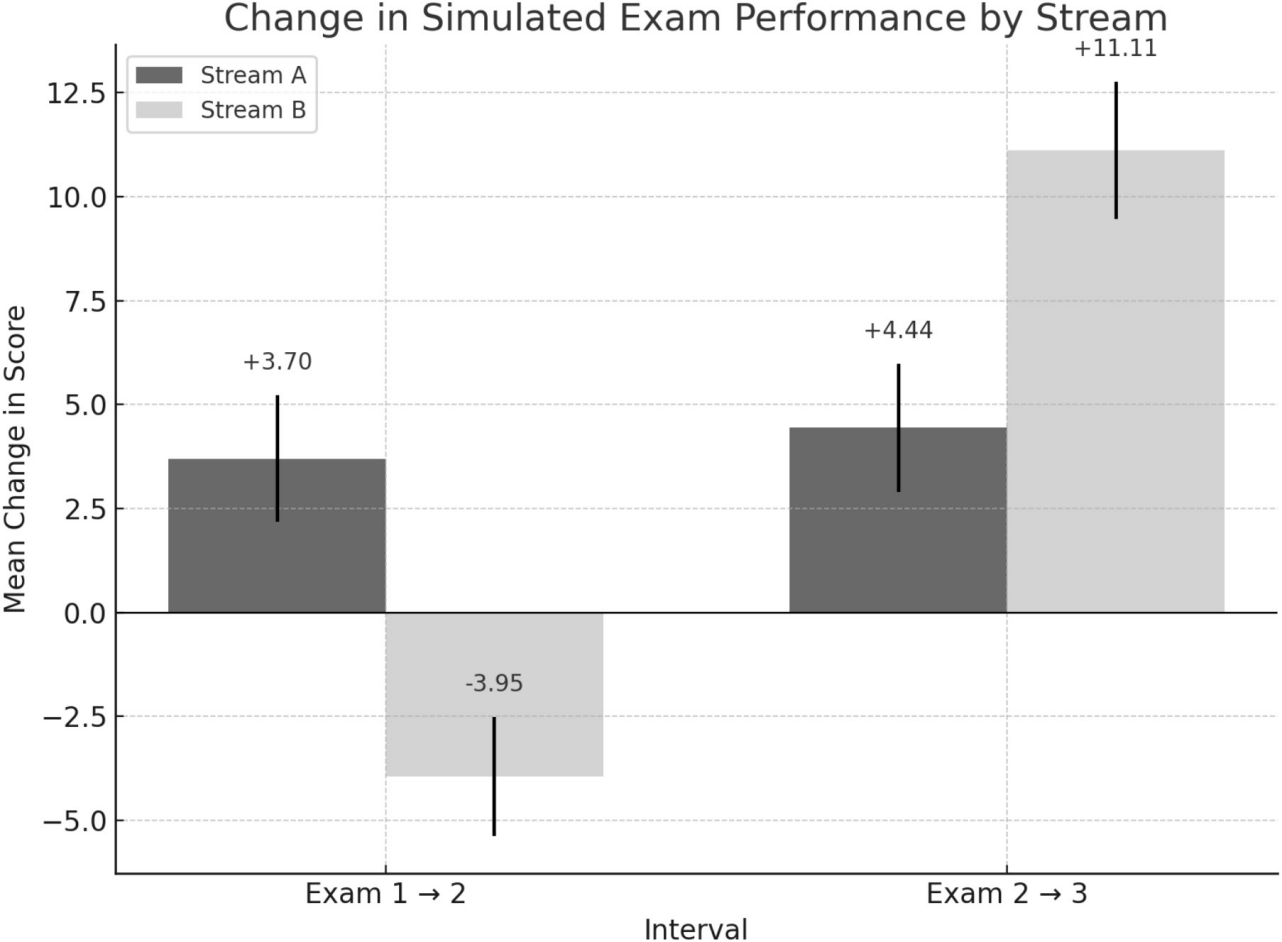
Change in simulated assessment performance by stream (error bars indicate 95% confidence interval). N.B. Stream A received teaching between Exams 1 and 2. Stream B received teaching between Exams 2 and 3.

Stream A steadily increased their score across exams, whereas Stream B demonstrated an initial decline followed by a marked improvement. Wilcoxon signed‐rank tests confirmed statistically significant changes at each interval (*p* < 0.001, *r* = 0.54–0.62). The most substantial gain was observed in Stream B between Exams 2 and 3 (+11.11) (Figure 2). Domain and clinical area based results can be found in Table 2. Lower‐performing students (< 58% in Exam 1) showed the greatest improvements—some exceeding 30 percentage points—whereas higher‐performing students (≥ 84%) demonstrated more modest gains, suggesting a ceiling effect. At the endpoint assessment, there was no significant difference between the mean scores of the respective streams.

**TABLE 2:** Improvements in prescribing performance in simulated assessments by PSA domain and clinical area.

PSA domain	Pre (%)	Post (%)	Δ %	Z	*p*	Effect size (*r*)
Prescribing	79.1	91.2	+12.1	−3.923	< 0.001	0.46
Prescription review	66.3	68.0	+1.7	−0.542	0.588	—
Planning management	56.4	60.8	+4.4	−1.193	0.233	—
Providing information	89.5	98.7	+9.2	−3.089	0.002	0.36
Calculations	30.1	68.2	+38.1	−5.869	< 0.001	0.68
ADRs	58.5	85.5	+27.0	−4.474	< 0.001	0.52
Drug monitoring	51.4	79.1	+27.7	−5.282	< 0.001	0.61
Data interpretation	47.3	84.5	+37.2	−5.337	< 0.001	0.62

Area‐specific simulated performance						
Area	Pre (%)	Post (%)	Δ %	Z	*p*	*r*
Medicine	70.3	78.5	8.2	−4.243	< 0.001	0.494
Elderly care	71.6	81	9.4	−3.855	< 0.001	0.448
Specialties (paediatrics/O&G/psych)	40.1	81.1	41	−7.201	< 0.001	0.837
GP	73.7	75	1.3	−0.116	0.908	—
High‐risk drugs	59.3	86.1	26.8	−7.41	< 0.001	0.861
Surgery	92.1	88.8	−3.3	−0.954	0.34	—
IV fluids	29	97	68	−5.29	< 0.001	0.615

**FIGURE 3:**
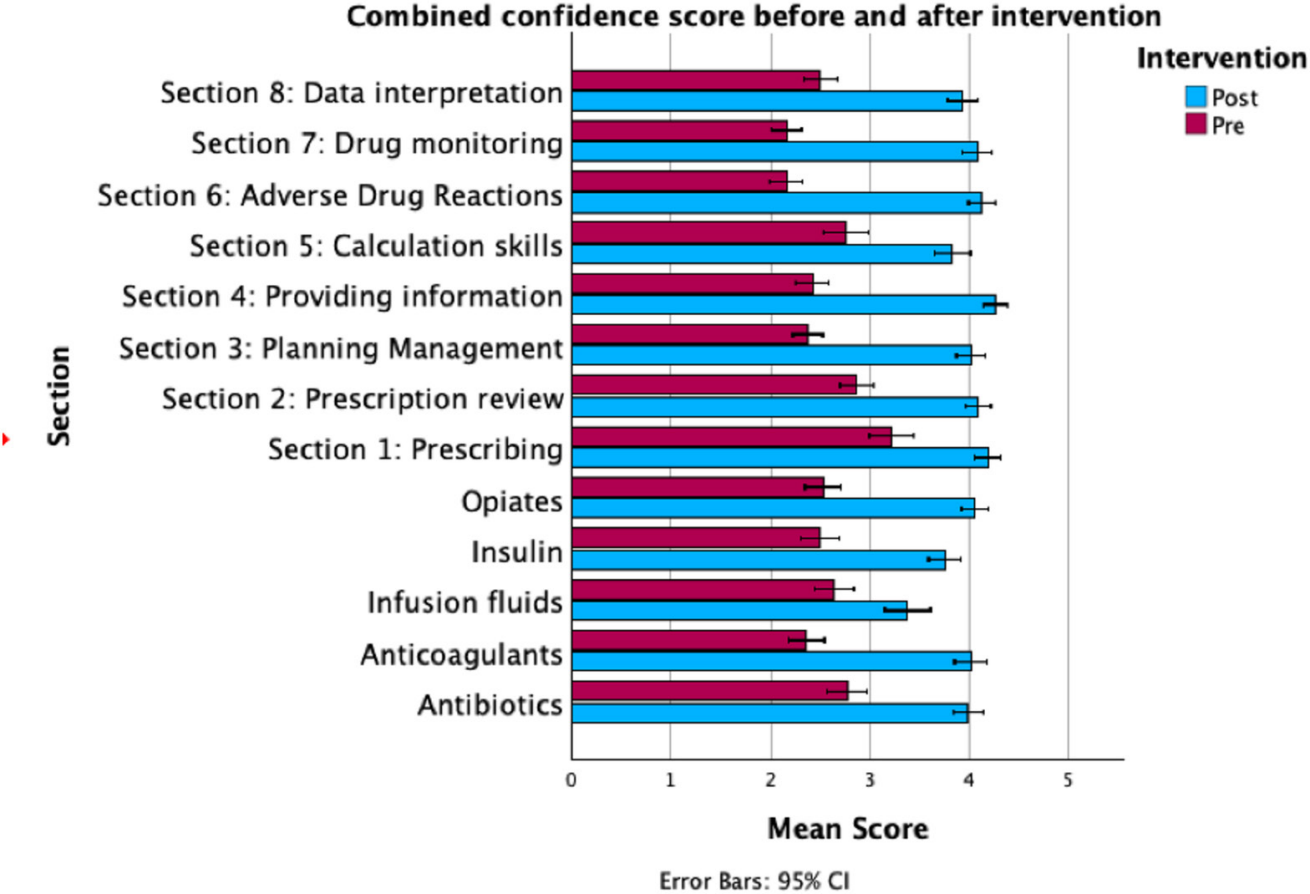
Pre‐ and post‐intervention confidence scores across PSA domains and high‐risk drug categories.

Domain‐specific gains were particularly evident in calculations, data interpretation and adverse drug reactions, with mean improvements exceeding 25%. Improvements across clinical prescribing areas (e.g., elderly care, specialities and high‐risk drugs) were also significant. Prescribing confidence, measured using a 5‐point Likert scale, increased across all PSA domains and high‐risk categories, with median scores rising by over one point post‐intervention (Wilcoxon *p* < 0.001 across all areas) (Figure 3).

*Domain‐specific gains were particularly evident in calculations, data interpretation and adverse drug reactions*.

### 3.2 National PSA Performance

Anonymised PSA scores were obtained for all final‐year students at WMS in 2023 for both participants and the non‐participant cohort. National PSA outcomes aligned with simulated assessment findings: Intervention participants achieved higher average scores than non‐participants in both January and March sittings, with 1.6% and 16.5% higher pass rates, respectively. The mean PSA scores for participants were 160 (January) and 145 (March), compared with 155 and 140 among non‐participants. Although causal conclusions are limited by potential confounding factors, the consistency of improvement across multiple performance metrics is encouraging.

### 3.3 Thematic Analysis of Interviews

**FIGURE 4:**
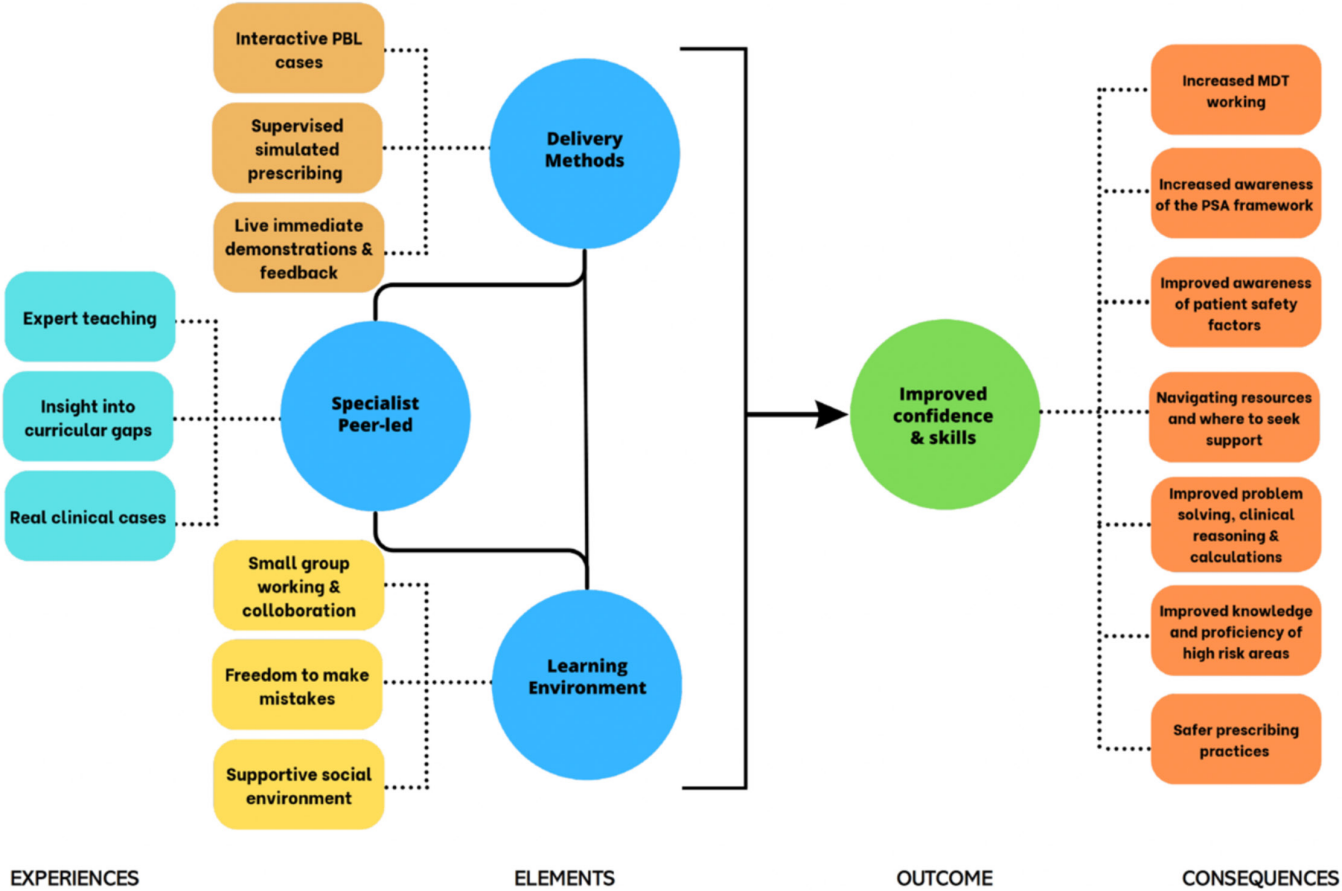
Visual summary of qualitative findings from thematic analysis.

Thematic analysis using Braun and Clarke's framework revealed four key themes: specialist peer insights, a supportive learning environment, dynamic delivery methods and improved confidence and skills. A model demonstrating how these factors in combination likely contributed to the success of this intervention is in Figure 4.

Students praised the value of learning from pharmacist peer tutors, noting their practical insights, relatability and shared academic background. This created a sense of openness and trust, encouraging students to engage and ask questions. As one participant explained, ‘pharmacist input is beneficial because they know the intricacies of certain drugs and interactions’, whereas another commented, ‘being peers puts them in a unique position; they understand our level of prescribing’. This familiarity helped to identify knowledge gaps and strengthened students' appreciation of pharmacists' roles within the wider multidisciplinary team. The peer‐led, small‐group format fostered a psychologically safe environment where students felt free to speak up. ‘I felt more comfortable asking questions’, one student noted, and another highlighted the benefit of immediate clarification: ‘You can clarify confusion immediately in a smaller group’. This setting was seen as a clear departure from traditional lecture‐based teaching.

*Pharmacist input is beneficial because they know the intricacies of certain drugs and interactions*.

Participants strongly preferred the dynamic, interactive delivery style, which included real‐life cases, whiteboard walkthroughs and simulated tasks. This approach improved knowledge retention and exam preparation. ‘The format—not just the content—made the course effective’, one student reflected, whereas another shared, ‘learning BNF navigation was invaluable; I'd have struggled in the PSA without it’. The programme also boosted students' confidence in prescribing, particularly for high‐risk scenarios. They reported feeling more prepared to prescribe independently and safely in clinical settings. ‘I feel confident prescribing in real life and doing it safely’, said one participant, whereas another stated, ‘I've learned to recognise drug interactions, like in AKI’.

Beyond teaching, students welcomed meaningful engagement with pharmacists, something they felt was lacking in traditional clinical placements. Several participants recommended formal integration of the course into the curriculum, citing its relevance, effectiveness and positive impact.

Overall, these qualitative findings reinforced the quantitative outcomes, highlighting the intervention's success in improving confidence, clinical readiness and support for pharmacist‐led peer education.

## 4 Implications

This pharmacist‐led, peer‐teaching innovation improved student prescribing confidence, performance and interprofessional awareness. It aligns with evidence supporting small‐group, case‐based teaching to enhance clinical competence [11, 12, 13], addressing longstanding gaps in undergraduate prescribing training [6, 8, 10]. Following a successful pilot, the programme was sustained for a third year at WMS, delivered by successive graduate‐entry medical cohorts. Key lessons included the value of cognitive congruence, the importance of simulation and feedback and the feasibility of low‐cost implementation using existing infrastructure [17, 18].

These insights have driven local curricular change. WMS is formally integrating the model into its prescribing curriculum. Teaching materials are standardised, tutor handovers formalised and structured peer tutor preparation introduced. Faculty are exploring funding options to recognise tutor time and ensure long‐term sustainability—an ongoing challenge in peer‐led models without institutional support [19, 20].

Although this iteration leveraged a unique pharmacist‐student cohort, the model could be adaptable, with junior doctors, pharmacists or teaching fellows delivering similar teaching elsewhere [19]. This would make the approach more sustainable; however, further research would be needed in these areas to assess if they retain the same benefits as a specialist peer‐led model. Sessions were scheduled outside core teaching hours, and faculty training comprised a single two‐hour orientation covering curriculum, case facilitation and marking standards. Implementation to date has been cost‐neutral beyond potential tutor reimbursement.

The programme has been extended to NHS trusts nationally, aiding international medical graduates preparing for the PSA. Its core components: Simulation, near‐peer delivery, structured feedback and clinical authenticity are transferable across contexts and professions [9, 18]. Similar approaches could enhance learning in domains including musculoskeletal medicine, clinical nutrition or specialist prescribing, offering a scalable, practice‐based approach to interprofessional, peer‐led simulation in healthcare education [20, 21].

## 5 Limitations

The single‐centre design and moderate sample size (*n* = 74) limit generalisability. Participants volunteered, introducing potential selection bias, with more motivated or confident students more likely to participate. The absence of demographic data prevented analysis of participant characteristics. Although participants outperformed non‐participants in the PSA, the lack of baseline prescribing data for non‐participants limits the ability to isolate the effect of the intervention. Comparing national PSA scores with volunteers who were not selected, rather than the full cohort, would have better addressed selection bias, as non‐volunteers may differ in motivation and engagement. Improvement in scores may also reflect repeated test exposure rather than the intervention alone. Interviewees' feedback may have been affected by social desirability bias. Furthermore, assessments were not externally validated, and overall assessment reliability scores were low; however, clinical skill area was assessed consistently and became more reliably measured over time. No long‐term follow‐up was conducted to assess skill retention over time or clinical impact.

Key lessons learnt

- Specialist peer teaching offers unique credibility and relatability not often seen in traditional faculty‐led formats
- Embedding simulated, clinical‐based prescribing tasks enhances student motivation and engagement
- Specialist peer‐led models should be extended across multiple disciplines and educational fields
- Interprofessional peer education can bridge theory and practice, particularly in clinical areas such as prescribing

## 6 Conclusion

This educational innovation demonstrates that pharmacist specialist peer‐led teaching is an effective and credible approach to enhancing prescribing education. It addresses national concerns about prescribing confidence and performance while offering broader lessons for curriculum designers seeking to leverage interprofessional expertise within student cohorts. By combining subject expertise with the benefits of peer learning, this model contributes to safer prescribing practices and more collaborative professional development. Similar innovations could be applied to other disciplines and encouraged by institutions; however, further research would be needed to assess whether they would retain the same benefits.

## Author Contributions

**Zain Mohammed:** conceptualization (equal), methodology (equal), investigation (equal), formal analysis (equal), data curation (equal), project administration (equal), resources (equal), software (equal), visualization (equal), validation (equal), writing – original draft (equal), writing – review and editing (equal). **Mohammed Sarwar Shah:** conceptualization (equal), methodology (equal), investigation (equal), formal analysis (equal), data curation (equal), project administration (equal), resources (equal), software (equal), visualization (equal), validation (equal), writing – original draft (equal), writing – review and editing (equal). **Imtanaan Abbas:** investigation (equal), resources (equal), data curation (supporting), validation (supporting), writing – review and editing (equal). **Nabeel Hussain:** investigation (equal), resources (equal), data curation (supporting), validation (supporting), writing – review and editing (equal). **Shehzar Shah:** investigation (equal), resources (equal), data curation (supporting), validation (supporting), writing – review and editing (equal). **Saira Chowdry:** investigation (equal), supervision (equal), validation (equal), resources (supporting), writing – review and editing (equal). **Shyam Balasubramanian:** investigation (equal), supervision (equal), validation (equal), resources (supporting), writing – review and editing (equal). **Kate Owen:** investigation (equal), methodology (equal), supervision (equal), validation (equal), writing – review and editing (equal).

## Ethics Statement

This study was approved by the Biomedical and Scientific Research Ethics Committee via the MBChB course delegated approval at the University of Warwick. Written informed consent was obtained from all participants.

## Conflicts of Interest

The authors declare no conflicts of interest.

## Supporting information


**Appendix S1:** Supporting Information.

## Data Availability

The data that support the findings of this study are available from the corresponding author upon reasonable request.
