# Supplementary material for: Pharmacist Peer‐Led Teaching Enhances Medical Undergraduate Prescribing: A Mixed‐Methods Study
Source: Clin Teach. 2025 Sep 9;22(5):e70192. doi: 10.1111/tct.70192 (PMC12421213; doi:10.1111/tct.70192)

Appendix 1: Qualitative results

### Sample sizes

A total of 80 students were recruited from a pool of 140 students. Six students withdrew from the study, leaving 74 participants. The distribution of participants across streams was as follows:

| Stream | Participants |
| --- | --- |
| Stream A | 36 |
| Stream B | 38 |
| Total | 74 |

# Assessment Performance

### Descriptive Statistics

#### Exam Performance (Raw Marks)

Overall Exam Performance

| **Statistic** | **Exam A** | **Exam B** | **Exam C** |
| --- | --- | --- | --- |
| Mean (Std. Dev) | 35.54 (6.01) | 35.19 (5.74) | 43.08 (4.01) |
| 95% Confidence Interval | 34.15 - 36.93 | 33.86 - 36.52 | 42.15 - 44.01 |
| Median | 36.00 | 35.00 | 44.00 |
| Range | 13 - 45 | 21 - 47 | 33 - 50 |
| Interquartile Range | 7 | 10 | 5.25 |

# Simulated Exam Performance by Group and Timepoint

| Exam | Stream A (Mean ± SD) | Stream B (Mean ± SD) | Overall (Mean ± SD) | Between group p-value (Mann-Whitney U) |
| --- | --- | --- | --- | --- |
| Exam 1 (Mock 1) | 33.97 ± 6.57 | 36.95 ± 4.78 | 35.54 ± 6.01 | 0.034 (Stream B > A) |
| Exam 2 | 37.67 ± 4.86 | 32.89 ± 4.93 | 35.19 ± 5.74 | <0.001 (Stream A > B) |
| Exam 3 (Final) | 42.11 ± 4.19 | 44.00 ± 3.57 | 43.08 ± 4.01 | 0.057 (n.s.) |

- Non-parametric tests were used due to non-normal distribution (Shapiro-Wilk p < 0.05).
- Within-group Friedman tests showed significant progression (p < 0.001) in both streams across all exams.


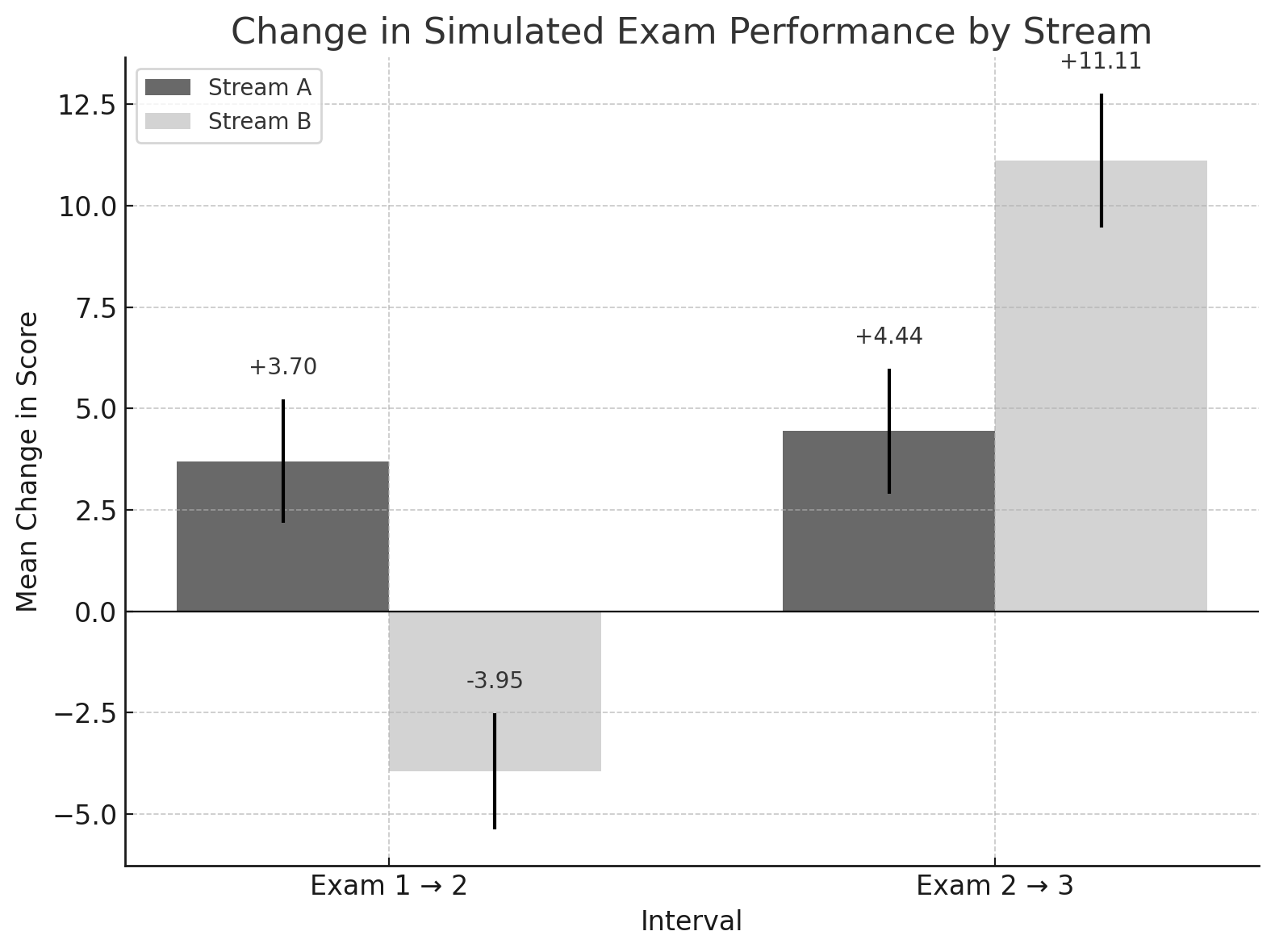


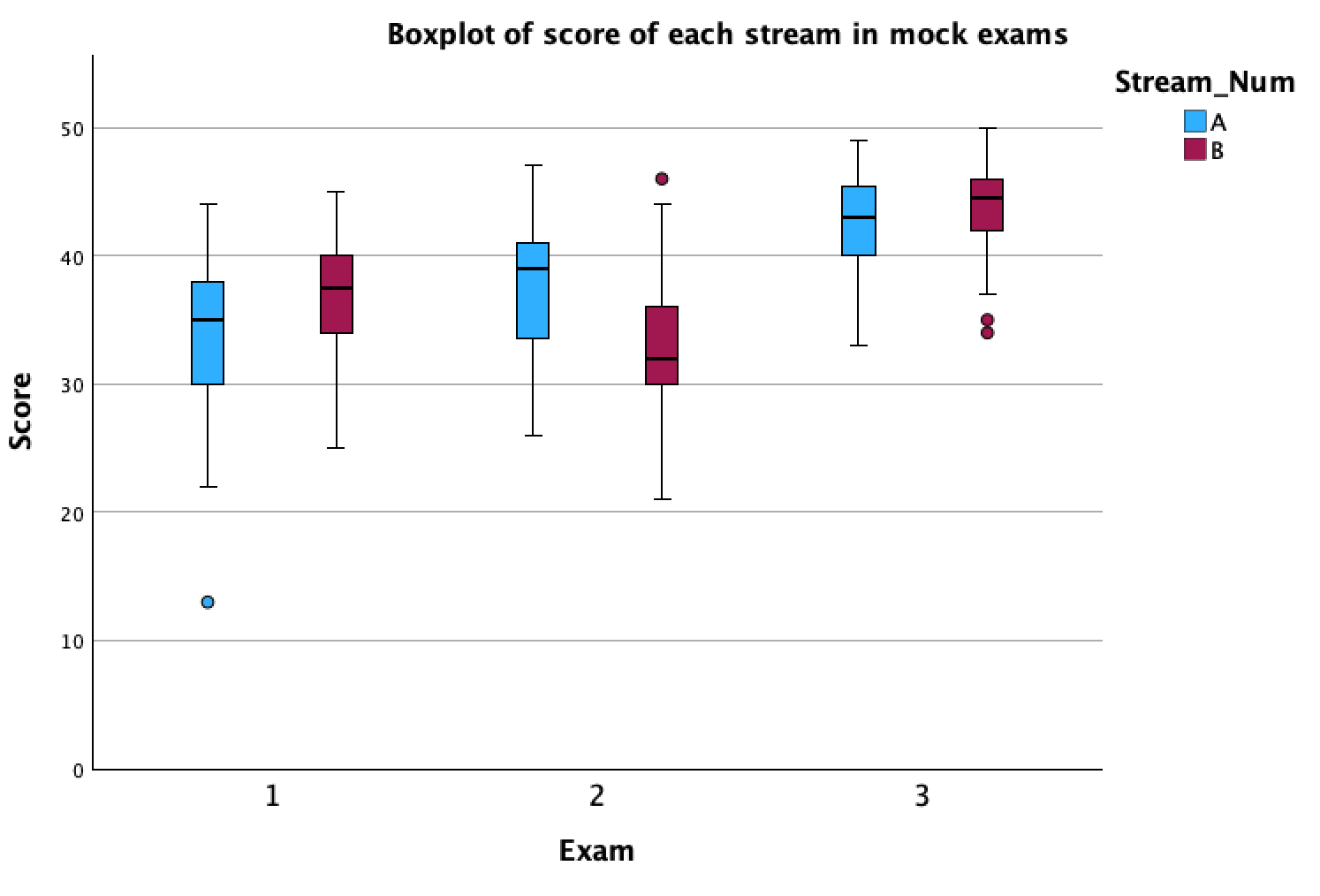


# Change in Performance Over Time

| Simulated Assessment performance | | | | |
| --- | --- | --- | --- | --- |
| Exam | Stream A (Mean ± SD) | Stream B (Mean ± SD) | Overall (Mean ± SD) | Between group p-value (Mann-Whitney U) |
| Exam 1 (Mock 1) | 33.97 ± 6.57 | 36.95 ± 4.78 | 35.54 ± 6.01 | 0.034 (Stream B > A) |
| Exam 2 | 37.67 ± 4.86 | 32.89 ± 4.93 | 35.19 ± 5.74 | <0.001 (Stream A > B) |
| Exam 3 (Final) | 42.11 ± 4.19 | 44.00 ± 3.57 | 43.08 ± 4.01 | 0.057 (n.s.) |
| Change in performance |  |  |  |  |
| Stream A |  |  |  |  |
| Interval | Mean Change (SD) | Z (Wilcoxon) | p-value | Effect Size (r) |
| Exam 1 → 2 | +3.70 (4.66) | -4.552 | <0.001 | 0.54 |
| Exam 2 → 3 | +4.44 (4.17) | -5.015 | <0.001 | 0.59 |
| Stream B |  |  |  |  |
| Interval | Mean Change (SD) | Z (Wilcoxon) | p-value | Effect Size (r) |
| Exam 1 → 2 | -3.95 (4.48) | -5.114 | <0.001 | 0.59 |
| Exam 2 → 3 | +11.11 (5.20) | -5.394 | <0.001 | 0.62 |

#### Exam Performance (in Percentages)

Table: Exam Performance Statistics and Participant Numbers

| **Statistic** | **Group/Stream** | **Exam A** | **Exam B** | **Exam C** |
| --- | --- | --- | --- | --- |
| Participants | Stream A | 36 | 36 | *36** |
|  | Stream B | 38 | 38 | 38 |
|  | Total | 74 | 74 | 74 |
| Mean (Std. Dev) | Overall | 71.08% (12.02%) | 70.38% (11.48%) | 86.16% (8.02%) |
|  | Stream A | 67.94% (13.13%) | 75.34% (10.07%) | 84.22% (8.40%) |
|  | Stream B | 74.06% (10.18%) | 65.68% (10.83%) | 88.00% (7.28%) |
| 95% Confidence Interval | Overall | 68.30% - 73.86% | 67.72% - 73.04% | 84.30% - 88.02% |
|  | Stream A | 63.50% - 72.38% | 71.92% - 78.76% | 81.38% - 87.06% |
|  | Stream B | 70.70% - 77.42% | 62.12% - 69.24% | 85.60% - 90.40% |
| Median | Overall | 72.00% | 70.00% | 88.00% |
|  | Stream A | 70.00% | 78.00% | 86.00% |
|  | Stream B | 75.00% | 64.00% | 89.00% |
| Range | Overall | 26% - 90% | 42% - 94% | 66% - 100% |
|  | Stream A | 26.00% - 88.00% | 52.00% - 94.00% | 66.00% - 98.00% |
|  | Stream B | 50.00% - 90.00% | 42.00% - 92.00% | 68.00% - 100.00% |
| Interquartile Range | Overall | 14% | 20% | 10.50% |
|  | Stream A | 16.00% | 16.00% | 11.11% |
|  | Stream B | 14.00% | 14.00% | 8.00% |

*Note: For Exam C, 28 students attended from Stream A. The data for the 8 students who did not attend was approximated by increasing their Exam B scores by the average change observed in their subgroup.

## Internal Consistency (Cronbach’s Alpha) by Domain

The following table summarises Cronbach’s Alpha for each prescribing domain across all three simulated exams. These values indicate acceptable to good internal consistency across most domains, improving from Exam 1 to Exam 3.

| Domain | Exam 1 | Exam 2 | Exam 3 |
| --- | --- | --- | --- |
| Prescribing | 0.702 | 0.735 | 0.743 |
| Prescription Review | 0.612 | 0.631 | 0.643 |
| Planning Management | 0.688 | 0.701 | 0.712 |
| Communicating Information | 0.677 | 0.689 | 0.708 |
| Calculation Skills | 0.575 | 0.603 | 0.632 |
| Adverse Drug Reactions | 0.694 | 0.718 | 0.726 |
| Drug Monitoring | 0.663 | 0.674 | 0.689 |
| Data Interpretation | 0.648 | 0.679 | 0.691 |
| Total for Assessment | 0.25 | 0.19 | 0.32 |

| Tests of Normality | | | | | | |
| --- | --- | --- | --- | --- | --- | --- |
|  | Kolmogorov-Smirnov^a^ | | | Shapiro-Wilk | | |
|  | Statistic | df | Sig. | Statistic | df | Sig. |
| Exam A | .140 | 74 | .001 | .945 | 74 | .003 |
| Exam B | .125 | 74 | .006 | .969 | 74 | .068 |
| Exam C | .145 | 74 | <.001 | .936 | 74 | <.001 |
| a. Lilliefors Significance Correction | | | | | | |

Note: Data was not normally distributed; hence, non-parametric tests were used.

### Between stream analysis (Mann-U Whitney)

Descriptive Statistics and Test Results

| **Final Score** | **Stream** | **N** | **Mean Rank** | **Sum of Ranks** |
| --- | --- | --- | --- | --- |
| Exam A | A | 36 | 32.08 | 1155.00 |
|  | B | 38 | 42.63 | 1620.00 |
|  | Total | 74 |  |  |
| Exam B | A | 36 | 47.00 | 1692.00 |
|  | B | 38 | 28.50 | 1083.00 |
|  | Total | 74 |  |  |
| Exam C | A | 36 | 32.63 | 1174.50 |
|  | B | 38 | 42.12 | 1600.50 |
|  | Total | 74 |  |  |

| **Test Statistic** | **Exam A** | **Exam B** | **Exam C** |
| --- | --- | --- | --- |
| Mann-Whitney U | 489.000 | 342.000 | 508.500 |
| Wilcoxon W | 1155.000 | 1083.000 | 1174.500 |
| Z | -2.114 | -3.709 | -1.905 |
| Asymp. Sig. (2-tailed) | 0.034 | <0.001 | 0.057 |

#### Summary of Results

The Mann-Whitney U test results indicated a statistically significant difference between streams A and B for Final Score A (U = 489.000, Z = -2.114, p = 0.034) and Final Score B (U = 342.000, Z = -3.709, p < 0.001). However, no significant difference was found for Final Score C (U = 508.500, Z = -1.905, p = 0.057).

Final Score A: Stream B had a significantly higher mean rank (42.63) compared to Stream A (32.08).

Final Score B: Stream A had a significantly higher mean rank (47.00) compared to Stream B (28.50).

Final Score C: No significant difference between Stream A (mean rank 32.63) and Stream B (mean rank 42.12).

### Within-Stream Analysis

#### Friedman test for Stream A

Stream A included 36 participants. The Friedman test results indicated a statistically significant difference in the mean ranks of the final scores within Stream A (Chi-Square = 41.239, df = 2, p < 0.001).

| **Final Score** | **Mean Rank** |
| --- | --- |
| Exam A | 1.28 |
| Exam B | 1.94 |
| Exam C | 2.78 |

| **Test Statistic** | **Value** |
| --- | --- |
| N | 36 |
| Chi-Square | 41.239 |
| df | 2 |
| Asymp. Sig. (2-tailed) | <.001 |

#### Friedman test for Stream B

Stream B included 38 participants. The Friedman test results indicated a statistically significant difference in the mean ranks of the final scores within Stream B (Chi-Square = 53.284, df = 2, p < 0.001).

| **Final Score** | **Mean Rank** |
| --- | --- |
| Exam 1 | 1.91 |
| Exam 2 | 1.22 |
| Exam 3 | 2.87 |

| **Test Statistic** | **Value** |
| --- | --- |
| N | 38 |
| Chi-Square | 53.284 |
| df | 2 |
| Asymp. Sig. (2-tailed) | <.001 |

# Change in Performance Over Time

Stream A:

| Interval | Mean Change (SD) | Z (Wilcoxon) | p-value | Effect Size (r) |
| --- | --- | --- | --- | --- |
| Exam 1 → 2 | +3.70 (4.66) | -4.552 | <0.001 | 0.54 |
| Exam 2 → 3 | +4.44 (4.17) | -5.015 | <0.001 | 0.59 |

Stream B:

| Interval | Mean Change (SD) | Z (Wilcoxon) | p-value | Effect Size (r) |
| --- | --- | --- | --- | --- |
| Exam 1 → 2 | -3.95 (4.48) | -5.114 | <0.001 | 0.59 |
| Exam 2 → 3 | +11.11 (5.20) | -5.394 | <0.001 | 0.62 |

Interpretation: Both groups showed net improvement. Stream B’s major gains occurred between Exam 2 and 3; Stream A improved steadily throughout.

# Domain-Specific Simulated Performance

| PSA Domain | Pre (%) | Post (%) | Δ % | Z | p | Effect Size (r) |
| --- | --- | --- | --- | --- | --- | --- |
| Prescribing | 79.1 | 91.2 | +12.1 | -3.923 | <0.001 | 0.46 |
| Prescription Review | 66.3 | 68.0 | +1.7 | -0.542 | 0.588 | - |
| Planning Management | 56.4 | 60.8 | +4.4 | -1.193 | 0.233 | - |
| Providing Information | 89.5 | 98.7 | +9.2 | -3.089 | 0.002 | 0.36 |
| Calculations | 30.1 | 68.2 | +38.1 | -5.869 | <0.001 | 0.68 |
| ADRs | 58.5 | 85.5 | +27.0 | -4.474 | <0.001 | 0.52 |
| Drug Monitoring | 51.4 | 79.1 | +27.7 | -5.282 | <0.001 | 0.61 |
| Data Interpretation | 47.3 | 84.5 | +37.2 | -5.337 | <0.001 | 0.62 |

#
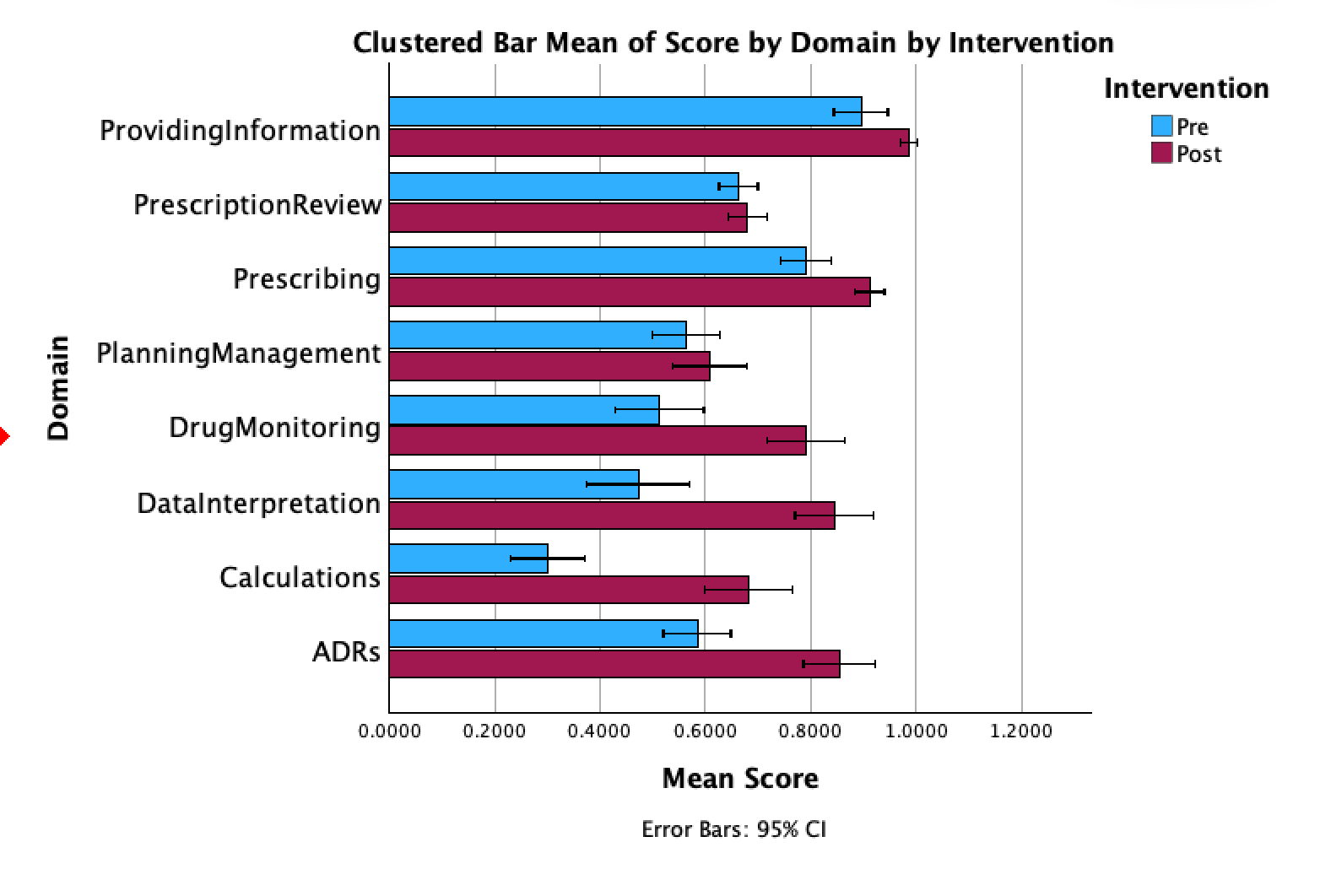


# Performance by Clinical Area

| Area | Pre (%) | Post (%) | Δ % | Z | p | **r** |
| --- | --- | --- | --- | --- | --- | --- |
| Medicine | 70.3 | 78.5 | +8.2 | -4.243 | <0.001 | 0.494 |
| Elderly Care | 71.6 | 81.0 | +9.4 | -3.855 | <0.001 | 0.448 |
| Specialties (Paeds/O&G/Psych) | 40.1 | 81.1 | +41.0 | -7.201 | <0.001 | 0.837 |
| GP | 73.7 | 75.0 | +1.3 | -0.116 | 0.908 | 0.013 |
| High Risk Drugs | 59.3 | 86.1 | +26.8 | -7.410 | <0.001 | 0.861 |
| Surgery | 92.1 | 88.8 | -3.3 | -0.954 | 0.340 | 0.111 |
| IV Fluids | 29.0 | 97.0 | +68.0 | -5.290 | <0.001 | 0.615 |


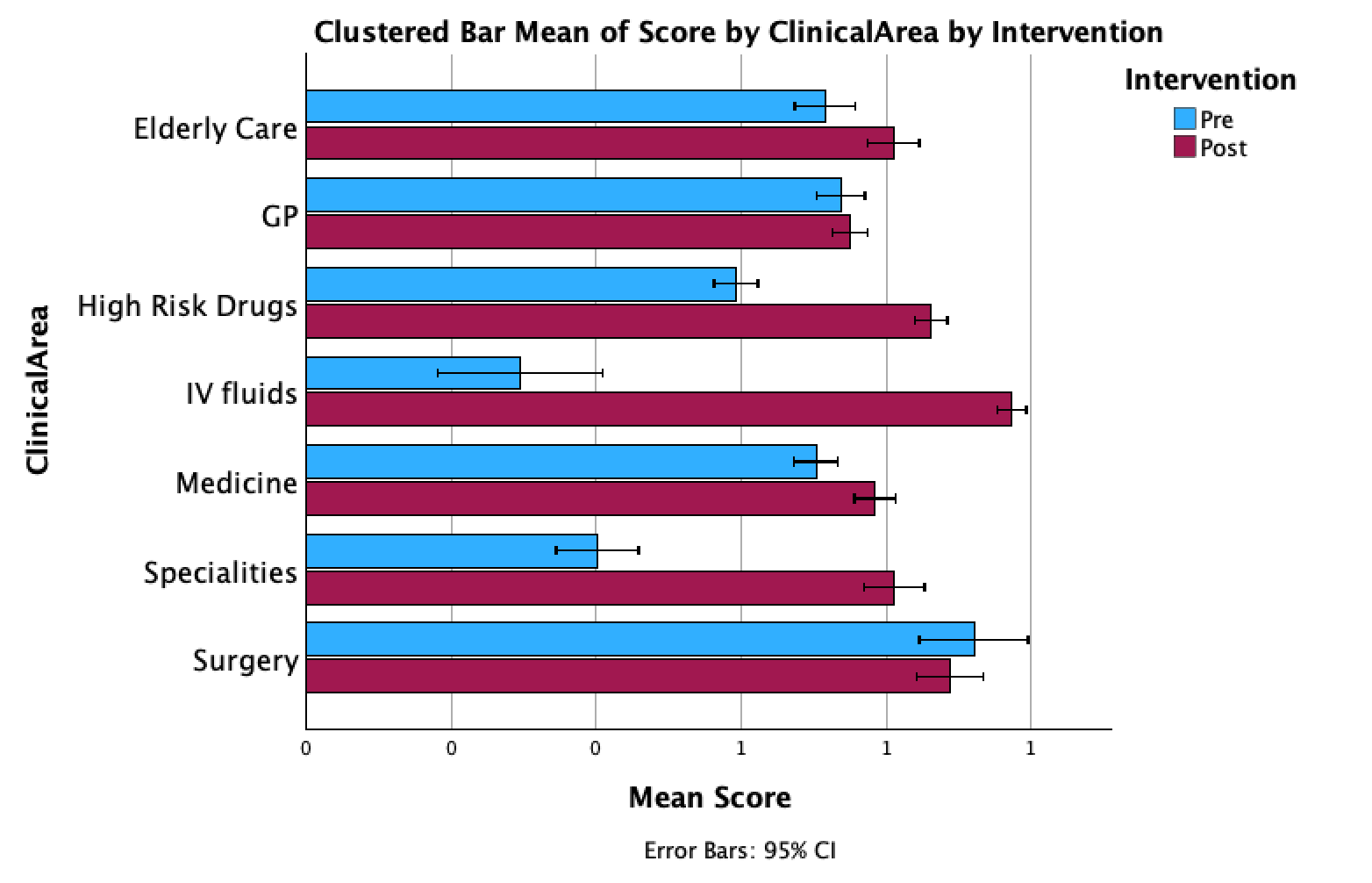


Actual PSA performance

|  | |  |  |
| --- | --- | --- | --- |
| **PSA Date** | | **02-Feb 2024** | **12-Mar 2024** |
| Number of students* | | 120 | 60 |
| Average marks per psa date | | 157 | 141 |
| Average marks per psa date excluding students on prescribing course | | 155 | 140 |
| Average marks per psa date for students on prescribing course only | | 160 | 145 |
| % pass rate per date | | 97.5% | 81.7% |
| % pass rate per date excluding students on prescribing course | | 96.9% | 77.3% |
| % pass  rate per date for students on prescribing course only | | 98.2% | 93.8% |
|  |  |  |  |

The PSA exam results indicate significant differences in performance between students who attended the prescribing course and those who did not. Among the 180 students who participated in the exams, those who attended the prescribing course demonstrated superior outcomes.

Specifically, students who attended the prescribing course achieved higher average marks: 160 compared to 155 for non-attendees in the first sitting and 145 compared to 140 in the second sitting. This positive impact is also evident in the pass rates, with students on the prescribing course achieving a pass rate of 98.2% in the first sitting and 93.8% in the second, compared to 96.9% and 77.3% for those who did not attend the course.

These findings suggest that the prescribing course significantly enhances student performance, resulting in higher average marks and pass rates across both exam dates. The data strongly supports the efficacy of the prescribing course as an intervention for improving PSA exam outcomes.

# Self-reported Confidence 1-5 Likert scale

| Pre/Post Confidence Scores | | | | |
| --- | --- | --- | --- | --- |
|  | **Mean (st dev)** | | **Wilcoxon Test Statistics** | |
| **Section** | **Pre-Intervention Confidence score** | **Post-Intervention Confidence score** | **Z** | **Asymp Sig 2 Tailed** |
| Section 1: Prescribing | 3.17 (0.996) | 4.21 (0.616) | -6.169 | <.001 |
| Section 2: Prescription review | 2.85 (0.76) | 4.1 (0.614) | -7.337 | <.001 |
| Section 3: Planning Management | 2.33 (0.622) | 4.06 (0.657) | -7.863 | <.001 |
| Section 4: Providing information | 2.37 (0.72) | 4.28 (0.546) | -7.963 | <.001 |
| Section 5: Calculation skills | 2.81 (1.057) | 3.87 (0.837) | -6.448 | <.001 |
| Section 6: Adverse Drug Reactions | 2.1 (0.736) | 4.16 (0.648) | -8.007 | <.001 |
| Section 7: Drug monitoring | 2.1 (0.686) | 4.12 (0.693) | -8.021 | <.001 |
| Section 8: Data interpretation | 2.47 (0.778) | 3.97 (0.71) | -7.410 | <.001 |
| Opiates | 2.48 (0.793) | 4.07 (0.629) | -7.760 | <.001 |
| Anticoagulants | 2.27 (0.773) | 4.01 (0.759) | -7.681 | <.001 |
| Insulin | 2.43 (0.861) | 3.77 (0.762) | -7.199 | <.001 |
| Antibiotics | 2.67 (0.9) | 4.01 (0.694) | -7.432 | <.001 |
| Infusion fluids | 2.62 (0.923) | 3.43 (1.035) | -5.183 | <.001 |

#
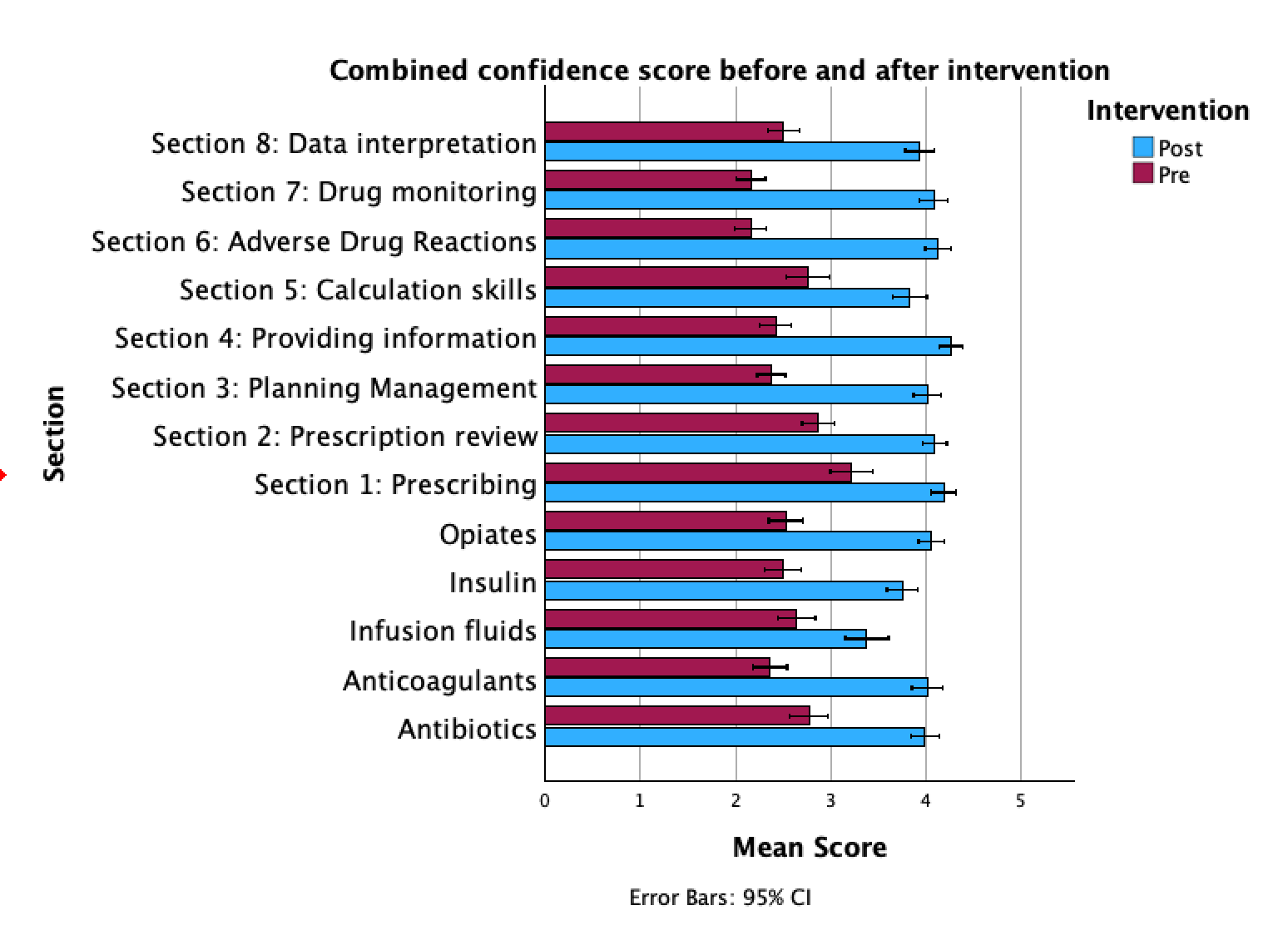

Supplement: Supplementary file 1 — Appendix S1: Supporting Information. [file TCT-22-e70192-s001.docx]
